# Estimating Upper Bounds for Occupancy and Number of Manatees in Areas Potentially Affected by Oil from the Deepwater Horizon Oil Spill

**DOI:** 10.1371/journal.pone.0091683

**Published:** 2014-03-26

**Authors:** Julien Martin, Holly H. Edwards, Florent Bled, Christopher J. Fonnesbeck, Jérôme A. Dupuis, Beth Gardner, Stacie M. Koslovsky, Allen M. Aven, Leslie I. Ward-Geiger, Ruth H. Carmichael, Daniel E. Fagan, Monica A. Ross, Thomas R. Reinert

**Affiliations:** 1 Florida Fish and Wildlife Conservation Commission, Fish and Wildlife Research Institute, St Petersburg, Florida, United States of America; 2 Patuxent Wildlife Research Center, United States Geological Survey, Laurel, Maryland, United States of America; 3 Department of Fish, Wildlife, and Conservation Biology, Colorado State University, Fort Collins, Colorado, United States of America; 4 Department of Biostatistics, Vanderbilt University, Nashville, Tennessee, United States of America; 5 Laboratoire de Statistique et Probabilités, Université Paul Sabatier, Toulouse, France; 6 Department of Forestry and Environmental Resources, North Carolina State University, Raleigh, North Carolina, United States of America; 7 Dauphin Island Sea Lab, Dauphin Island, Alabama, United States of America; 8 University of South Alabama, Mobile, Alabama, United States of America; 9 Sea to Shore Alliance, 4411, Sarasota, Florida, United States of America; Texas A&M University-Corpus Christi, United States of America

## Abstract

The explosion of the Deepwater Horizon drilling platform created the largest marine oil spill in U.S. history. As part of the Natural Resource Damage Assessment process, we applied an innovative modeling approach to obtain upper estimates for occupancy and for number of manatees in areas potentially affected by the oil spill. Our data consisted of aerial survey counts in waters of the Florida Panhandle, Alabama and Mississippi. Our method, which uses a Bayesian approach, allows for the propagation of uncertainty associated with estimates from empirical data and from the published literature. We illustrate that it is possible to derive estimates of occupancy rate and upper estimates of the number of manatees present at the time of sampling, even when no manatees were observed in our sampled plots during surveys. We estimated that fewer than 2.4% of potentially affected manatee habitat in our Florida study area may have been occupied by manatees. The upper estimate for the number of manatees present in potentially impacted areas (within our study area) was estimated with our model to be 74 (95%CI 46 to 107). This upper estimate for the number of manatees was conditioned on the upper 95%CI value of the occupancy rate. In other words, based on our estimates, it is highly probable that there were 107 or fewer manatees in our study area during the time of our surveys. Because our analyses apply to habitats considered likely manatee habitats, our inference is restricted to these sites and to the time frame of our surveys. Given that manatees may be hard to see during aerial surveys, it was important to account for imperfect detection. The approach that we described can be useful for determining the best allocation of resources for monitoring and conservation.

## Introduction

Knowledge about the occurrence of protected species, populations and communities in areas affected by man-made or natural disasters (e.g., oil spills, release of other hazardous materials, hurricanes) is important from both policy and management standpoints [1]. In particular, the occupancy rate (the proportion of sites occupied by a species) and estimates of abundance can be used to help resource managers respond to a disaster in ways that most effectively mitigate its consequences [2]–[4]. Surveys of large areas are often conducted from aircraft, and, if the surveys are appropriately designed, the data collected can be used to infer the distribution of the organisms of interest. There are at least two major difficulties estimating probability of occurrence (the probability that a species is present at a site) and abundance from aerial survey data: (1) the area of interest (e.g., total area of the disaster zone) is generally too large to be covered entirely; and (2) only a portion of the animals of interest in the covered area are observed because of imperfect detection. In the case of marine mammals, some animals may go undetected because they are underwater and cannot be seen by the observer, while others may be at the surface but nevertheless are missed by the observer [5], [6]. Random sampling and the application of recently developed site occupancy and abundance models can be used to address these problems; however, in the case of monitoring rare or elusive species, a risk of implementing such protocols is that no organisms will be detected.

Until recently, no method had been developed to estimate occupancy rate when no animals were detected during a survey [3], [7]. Thus, many managers and policy makers tasked with assessing damage in disaster impacted areas resort to exhaustive searching in an attempt to cover as much area as possible, sometimes without following a statistically rigorous sampling design. In most situations, exhaustive searching is infeasible, and target species can easily be missed [5], [6], [8]. A recently developed statistical approach, however, can be used to estimate the probability of occurrence and occupancy rates of rare or elusive species, even when no organisms are detected during a dedicated survey, as long as some prior information on the presence of the species in the area of study is available [3]. This approach can help resource managers improve the survey protocols they use to respond to disasters. Here, we applied this new methodology to estimate occupancy rate of Florida manatees (*Trichechus manatus latirostris*) in the areas of Florida, Mississippi and Alabama affected by the Deepwater Horizon Gulf of Mexico oil spill. The conditional occupancy estimator, in combination with the use of prior information on detection probability, allowed us to estimate the occupancy rate of manatees, although no manatees were observed in our sampled areas. We extended this approach to derive an upper estimate for the number of manatees potentially present in the area impacted by oil at the time of our surveys.

In the context of scenario planning [1], estimates of potential damage are often needed, even when information available to the policy makers is incomplete or missing. The Bayesian approach that we have developed can be used to derive useful estimates by combining and borrowing information from different data sources (e.g., empirical information from the study area, relevant information from contiguous sites and from the published literature). This information is relevant to conservation and should be useful for planning wildlife monitoring of rare or elusive species.

The explosion of British Petroleum's Deepwater Horizon MC252 offshore drilling platform (April 2010) in the Gulf of Mexico created the largest marine oil spill in U.S. history. Over 84 days, the damaged well discharged an estimated 7×10^5^ m^3^ of crude oil [9] into the Gulf of Mexico impacting marine and coastal habitats in Louisiana, Mississippi, Alabama and Florida that are used by many species of plants, birds, reptiles and mammals [10], including the Florida manatee Trichechus manatus latirostris, which is listed as endangered by the U.S. Fish and Wildlife Service (FWS) [11]. Florida manatees are large herbivorous aquatic mammals, most commonly found in fresh and brackish rivers, bays, and estuaries in the subtropical regions of Florida and the southeastern United States. Although most of the Florida manatee population is found in peninsular Florida, manatees have also been reported to seasonally occupy Louisiana, Mississippi, Alabama and the Florida Panhandle in habitats reported to have been affected by the Deepwater Horizon oil spill [12],[13]. The U.S. Congress established the Natural Resource Damage Assessment (NRDA) provisions of the Oil Pollution Act to respond to oil spills and allow restoration of injured or destroyed natural resources [1]. Our investigation was conducted as part of the NRDA process to examine the potential for impact to manatees from the Deep Water Horizon oil spill.

To document the presence and numbers of manatees in areas affected by oil, we developed a random sampling aerial survey design that could be used for estimating abundance of aquatic mammals. Our survey design was based on long-established, fundamental survey design principles and innovative modeling techniques [2], [8], [14]. As demonstrated here, this design can provide useful results even in instances when no animals are observed in sampled plots during the surveys. Manatees can be exposed to oil in many different ways, for instance through their skin, gastrointestinal, or respiratory tracts. In fact, exposure to toxic compounds could occur even after the well was capped (e.g., through ingestion, or respiration of fumes). We did not attempt to quantify the magnitude of the impact or exposure; instead, our aim was to obtain an upper estimate for the number of manatees that may have been present in suitable manatee habitats potentially impacted by oil exposure during our surveys.

## Materials and Methods

### Study Area and Sampling in Areas Impacted by the Oil Spill in 2010

**Figure 1:**
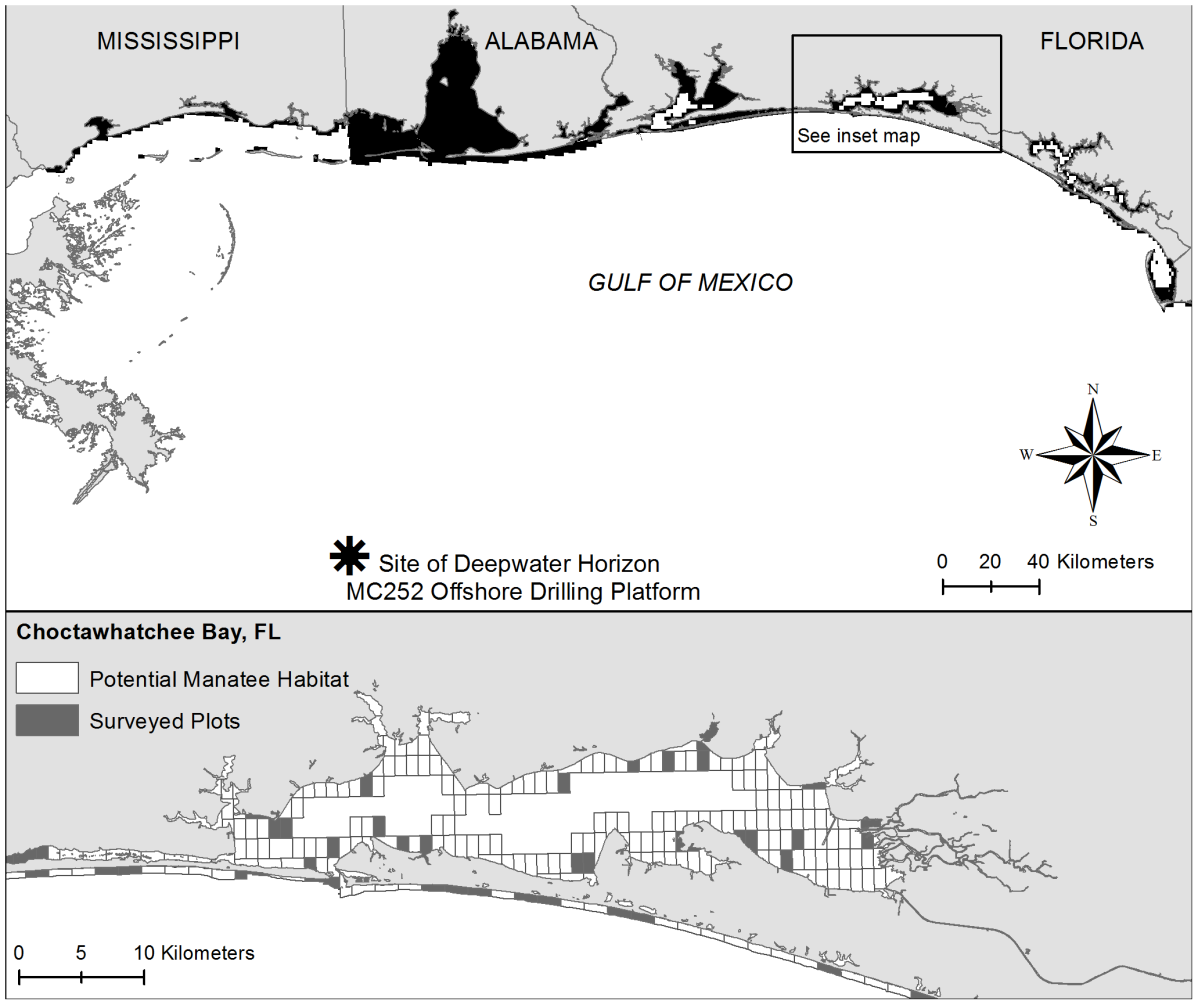
Areas surveyed in helicopters to obtain the upper estimate of the number of manatees in areas potentially affected by the Deepwater Horizon oil spill.

We conducted aerial surveys between September 12–16, 2010 to estimate manatee occupancy in the coastal regions of Mississippi, Alabama and the Florida Panhandle (hereafter referred as regions). Alabama and Mississippi were surveyed between September 12–13, and Florida between September 14–16. Work on this project was conducted under USFWS research permit #MA773494-10. The survey included areas that were reported to have been potentially impacted by the Deepwater Horizon oil spill. Sampled plots considered in this study included only areas that were deemed likely manatee habitats (based on bathymetry of less than 3.7 m and the presence of seagrass). The study area (total potential manatee habitat) was divided into 1,778 plots (each ∼1.3 km^2^, Fig. 1). The total number of plots per region (*T*, for Florida, Alabama and Mississippi) was 1,006 for Florida (13% of the total number of sites in Florida were surveyed, see Table 1), 400 for Alabama (10% were surveyed) and 372 for Mississippi (10% were surveyed) (Table 1). In each state or region, *J* plots were randomly selected for sampling (Table 1, Fig. 1). Manatees were counted during two consecutive helicopter surveys at each plot (using the same survey protocols for altitude [750 ft] and speed [80 kts] that are commonly used for fix-winged aircraft); each survey of a plot took approximately 2 minutes. Each helicopter included two observers and a pilot. Although no manatees were sighted during the on-effort portions of the survey, manatees were known to be present on or within a few days of the oil spill surveys based on telemetry records from one GPS-tagged animal, one off-survey aerial sighting of seven animals, and three citizen-reported sightings verify the presence of manatees within unsurveyed plots on the day of the survey in Alabama. In addition, one GPS-tagged animal occupied a surveyed plot on the survey day, but not at the same time the plot was surveyed and one manatee was sighted in Mississippi on September 14, and another was seen in Florida on September 15 in an unsurveyed plot. (Pers. Comm. R. Carmichael, K. Rigney).

**Table 1:** Estimates of occupancy rate (γ) of manatees in three regions (Florida, Alabama and Mississippi) that were affected by the Deepwater Horizon oil spill.

	Florida	Alabama	Mississippi
Sampled (*J*)	133	40	37
Total (*T*)	1006	400	372
Occupancy (γ)	0.005 [0.001–0.024]	0.025 [0.003–0.08]	0.027 [0.003–0.08]

The number of sampled plots (*J*) and the total number of plots for each region are also reported (*T*).

### Study Area and Sampling in West Florida in 2011

To estimate detection probabilities and derive an upper estimate of the number of manatees in Florida, we used an additional set of surveys (see section titled: “Estimation of Detection Probability”). We used a similar survey method as the one described above, except that the surveys were conducted from February 28 to March 21, 2011. Estuaries, rivers, creeks and coastlines from 26 counties (from Escambia to Monroe) on the west coast of Florida were surveyed with fixed-wing aircraft. Each plane included two observers and a pilot.

### Statistical Analyses

#### Occupancy Rate in Areas Impacted by the Oil Spill

We applied a model that was described by Dupuis et al [3] (hereafter referred as Dupuis's model) to estimate the occupancy rate of manatees in each region (γ):
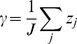
(1)where *z_j_* is the indicator of the presence of manatees in plot *j* (*z_j_* = 1 if at least one manatee is present in plot *j*; and 0 otherwise). Therefore, the occupancy rate (γ) can be interpreted as the proportion of sites occupied by manatees. The estimation of the occupancy rate is conditioned on the presence of the species of interest, information which can be obtained from auxiliary sources (see justification for assumed presence of manatees in regions in the section “Study Area and Sampling in Areas Impacted by the Oil Spill”). This conditioning allows us to obtain an estimate of occupancy rates even in the case that no detection occurred during the sampling period of the survey. To obtain unbiased estimates of occupancy, it is important to account for probability of detection *q*, where *q* is the probability of detecting at least one manatee at a sampled plot given that it was present. For a detailed description of the likelihood functions used in Dupuis's model, see [3]. We estimated *q* with the method described below and used the estimate of *q* as an informative prior in Dupuis's model.

#### Estimation of Detection Probability

Because no manatees were detected in the sampled plots in the areas impacted by the oil spill, it was not reasonable to estimate *q* directly in the sampled areas. Therefore, we applied a Bayesian formulation of occupancy models to estimate *q* from data that were available for the west coast of Florida in 2011 (see “Study Area and Sampling in West Florida in 2011”). In this model, we assumed that detection data for each plot *j* during the survey *t* followed a Bernoulli distribution:
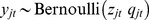
(2)where *z_jt_* is the state of occupancy in plot *j* during survey *t* and *q_jt_* is the detection probability of the manatee in this plot during this survey. We defined the occupancy state as *z_jt_*∼Bernoulli (φ*_jt_*), where φ*_jt_* is the occurrence probability (i.e., the probability that site *j* is occupied by at least one animal [2]). Parameters φ*_jt_* and *q_jt_* can be modeled as a function of covariates using a logit link:
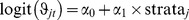
(3)

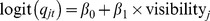
(4)where the strata covariate could take the values 1 or 0; 1 for plots in which manatees were expected to have a lower probability of presence (because of habitat features), and 0 in plots with more desirable habitat features for manatees. Visibility was also a binary variable with 0 meaning high visibility and 1 meaning poor visibility.

To estimate detection probabilities, we used equations 2, 3, and 4 with coefficients estimated from west Florida to predict *q_jt_* for each site, and then compute the mean *q_jt_* for each region (Florida, Alabama, and Mississippi). This approach allowed us to account for region specific detection probabilities, which varied among regions because of visibility. The detection/non detection data (eqn 2) included the observations of both observers; in other words, the data were pooled across observers within each survey. For instance, a plot was considered occupied if at least one manatee was reported at a plot by at least one of the two observers

#### Upper Estimate for the Number of Manatees

We derived an upper estimate for the number of manatees (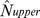) in Florida with the equation below:
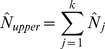
(5)

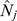
 is the abundance at each occupied site *j*. *k* was set to the upper 95%CI of the number of sites occupied and was derived from estimates obtained from Dupuis's model [3] (see eqn 7).

To derive the upper estimate for the number of manatees, 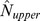, we assumed that the number of manatees at each site *j* followed a Poisson distribution:
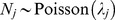
(6)
*k* was set to the upper 95%CI of the number of sites occupied derived from Dupuis's model:
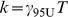
(7)γ_95U_ corresponds to the upper 95%CI of the occupancy rate estimated by Dupuis's method [3], and *T* is the total number of sites in the region of interest. In Equation 6, *λ_j_* corresponds to the mean number of manatees in an occupied site. *λ_j_* was estimated with a zero-truncated beta-binomial mixture model described in detail by [8], in which abundance per occupied site followed a zero-truncated Poisson distribution, and the observed counts *C_jt_* followed a beta-binomial distribution:
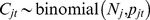
(8)where *p_jt_*∼beta(α, β). The count *C_jt_* was obtained from the primary observers (usually the more experienced observer), at sites adjacent to the areas potentially affected by the oil spill in Florida. For this purpose, we used counts obtained between Franklin County and Pasco County located on the west coast of Florida (see “Study Area and Sampling in West Florida”). This approach accounts for sources of heterogeneity or non-independence in detection probabilities [8]. Ideally, we would have estimated *p_jt_* using the repeated counts from the adjacent area, but the data were too sparse for the model to return estimates of *p_jt_*. Thus, we used the values of α and β based on the study published by Martin et al. [8]. The derived estimate of *p_jt_* was 0.56, which appeared reasonable for the present problem and represented the best available published scientific information. This estimate of 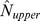 was conditioned on the upper 95%CI value of the occupancy rate. We only computed 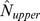 for Florida because the count data in the area adjacent to the zone potentially impacted by the oil spill were in Florida, and we did not think that these data were as representative of Alabama and Mississippi. Nevertheless, we computed estimates of occupancy rates for Alabama and Mississippi because they provide new information about the occupancy in these states.

#### Estimation Methods and Software

We fitted N-mixture and occupancy models using the Bayesian approach and Markov chain Monte Carlo (MCMC) simulation methods with program WinBUGS 1.4 [15]. We ran three chains with initial values picked randomly from their priors for each parameter. We assessed the chains' convergence to their stationary distributions using the Brooks-Gelman-Rubin diagnostic [16]. Dupuis's model was fitted using a Bayesian approach and Markov chain Monte Carlo (MCMC) simulation methods to estimate the occupancy rate γ*_r_*, using the MatLab codes provided in Bled et al. [7]. For the sake of our analysis, the most relevant parameter was the 95% upper credible interval of γ*_r_*, which can be viewed as the upper limit of proportion of sites occupied with a probability of 0.95.

## Results

The Bayesian estimate of the occupancy rate for Florida was 0.005, ranging from 0.001 to 0.024 with a probability of 0.95. Results for the other states (Alabama and Mississippi) are presented in Table 1. When no animals are observed in the sampled plots, this approach is useful in identifying the upper bound of the proportion of sites the species occupied. In the case of Florida, the 95% credible interval indicates we are confident with a high probability that fewer than 2.4% of the sites that include manatee habitat could have at least one manatee in them. Thus, probably fewer than 24 plots (*k*∼0.024×1006, see eqn 7, where γ_95U_ = 0.024, and *T* = 1006 in Florida) are expected to be occupied in Florida.

**Figure 2:**
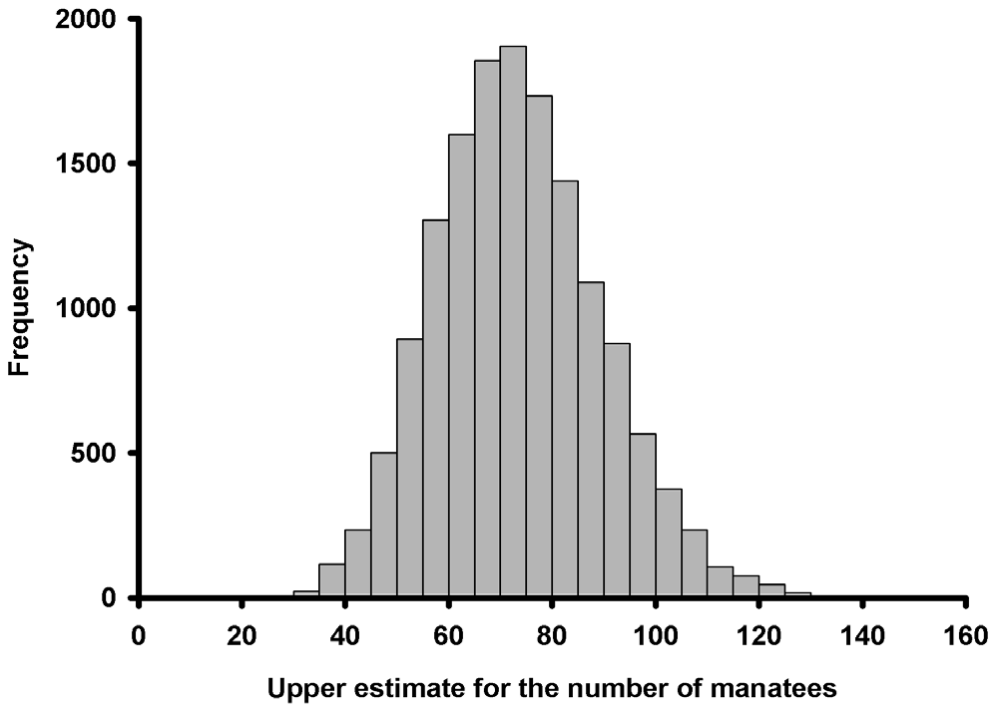
Posterior distribution of the upper estimate for the number of manatees in Florida (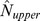) in manatee habitats potentially affected by the Deepwater Horizon oil spill during the surveys.

Because we used a conditional occupancy approach, we assumed that at least one manatee was present in each region, which is why the lower bounds of the 95%CI remain greater than 0. The upper 95%CI for Alabama and Mississippi were greater than for Florida, not because occupancy was necessarily greater but simply because a smaller number and proportion of plots were surveyed in Alabama and Mississippi. This point emphasizes the importance of interpreting the 95%CI of the occupancy rate with caution, because as the proportion of sites surveyed increases, the precision of the estimate also increases. Although occupancy rate is a useful and increasingly used parameter in wildlife studies, in our case, an upper estimate of the number of animals, 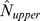, was desirable for evaluating the potential impact of the oil spill on manatees. To derive an upper estimate for the number of manatees present in the areas affected by the oil spill in Florida, we used estimates of manatee abundance per plot from data collected in habitats that were similar (e.g., in terms of bathymetry and distance to seagrass beds) and adjacent to the areas impacted by the oil spill (see Methods for details about the model used to derive the estimate). The posterior mean of 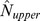 was 74 manatees, with a 95% interval of 46 to 107 (Fig. 2). This estimate of 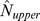 was conditioned on the upper 95%CI value of the occupancy rate. The variation in the estimate of maximum abundance reflects the uncertainty about the abundance per occupied plots and accounts for imperfect detection associated with our counts of manatees in the sites adjacent to the areas potentially impacted by the oil spill in Florida. The lower 95%CI is greater than 0 because 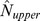 is an upper estimate for the number of manatees (furthermore, we are also assuming that at least one manatee is present in each of the states considered); in our case, the estimator uses the upper value of the 95%CI of the occupancy rate, i.e., 0.024 for Florida. Nevertheless, our results are not necessarily inconsistent with the hypothesis that there were no additional manatees to the ones that were reported or assumed to be there at the time of the surveys.

## Discussion

Our analyses were motivated by the NRDA process to investigate the potential for impact to manatees from the Deepwater Horizon oil spill [1]. We estimated that fewer than 2.4% of manatee habitats in our area of inference in Florida may have been potentially occupied by manatees (Fig. 1). We also estimated that 107 or fewer live manatees may have been present in that same area in Florida (Fig. 1) during the time of our surveys, which were conducted after the oil spill. Our analyses represent a snapshot of the conditions present during the time the surveys were conducted. We computed the upper estimate for the number of manatees for Florida only, because the count data were collected in Florida, adjacent to the zone potentially impacted by the oil spill, and we did not think that these data were as representative of Alabama and Mississippi. On the other hand, because, we used the same visibility criteria for each surveyed plot, we believe the detection probability that we used is reasonably representative of the areas considered.

A few issues are worth pointing out. Although, our upper estimate for the number of manatees potentially present appears reasonable, this estimate is tied to the time and area of inference. In our case, we obtained an estimate that represents a snapshot of the situation, post oil spill. Because we restricted our analyses to habitats considered likely manatee habitats (based on a bathymetry of less than 3.7 m and the presence of seagrass), our inference is also restricted to these sites. We do not claim that our estimates should be interpreted as the maximum number of manatees potentially affected by the oil spill (as noted above these estimates are tied to the time and area of inference); instead, our estimates reflect the upper bound of the number of manatees that may have been present at the time of the survey and in the surveyed areas. Additionally, this estimate does not account for manatees that could potentially have died from the spill. Another issue not addressed by our study is the possibility that manatees potentially affected by the oil spill could have left the area before our surveys. Of course, the surveys could be adapted to adjust the temporal scale of inference. Obviously borrowing information from contiguous sites (e.g., average number of animals per occupied plot) is less desirable than using direct information from the sites of interest. However, one key point of our work is to show that it is possible to extract useful information from wildlife surveys, even if no animals have been observed in sampled areas, as opposed to simply resorting to guess work or resigning ourselves to complete ignorance. Indeed, in many situations in which decisions have to be made, managers and biologists often resort to “best guesses” or intuition. Here, we provide a methodology to improve “best guess” estimates (e.g., solely based on expert opinions) by synthesizing the best information available from aerial surveys (empirical or published) into a Bayesian analysis.

Finally, if no manatees are observed, as the number of sampled plots increases, the estimate of occupancy rate should tend toward 1/*T* (it is 1 over *T* because the model assumes that at least one manatee is present in the study area. Note that the values of lower 95%CI in Table 1 are close to 1/*T*). In addition, the precision of the estimate also increases with the number of plots sampled. For instance, the upper 95%CI for the occupancy rate in Alabama was greater than the estimate in Florida, not necessarily because of a difference in occupancy, but simply because a larger proportion of plots were surveyed in Florida. Thus, if the number of plots surveyed was higher in Alabama but no manatees were observed in these additional surveyed plots, the upper 95%CI would go down for Alabama. Similarly, if we had increased the number of surveyed plots in Florida but no manatees were observed in the sampled plots, both the upper 95%CI for the occupancy rate and the derived estimate 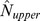 would go down. This makes logical sense, as we increase the proportion of sites surveyed and no animals are observed, we are gaining more confidence that fewer sites are occupied. As a consequence, everything else being equal, the derived upper estimate for the number of manatees potentially present would also go down. Note that if animals are observed in the sampled plots, then traditional occupancy models and N-mixture models can then be applied to obtain estimates of occupancy and abundance.

One application of our approach is that it can be used for contingency planning for potential rescues of animals impacted by a catastrophe or to gauge the potential for future impacts to a population. In other words, by obtaining an estimate of the maximum number of animals potentially present in a disaster zone, policy makers can better allocate resources necessary for responding to the catastrophe. Additionally, we recommend this as a decision support tool for wildlife monitoring of abundance or occupancy. For instance, if a pilot study indicates that the maximum number of animals potentially present in a large area represents a negligible fraction of the overall population, biologists may decide not to allocate limited resources to aerial monitoring of that area. In the case of our Florida study, we found that fewer than 2.4% of likely manatee habitats were likely to be occupied, and this estimate could be used as prior information for the design of future studies in the Florida Panhandle. Similarly, the estimates of occupancy rates for Alabama and Mississippi can be used as prior information for the purpose of designing future aerial surveys in these areas (Table 1).

Our model allowed us to borrow information from different sources of data and to propagate uncertainty. As noted earlier, to improve scenario planning and response to disasters, it is often necessary to obtain estimates of potential damage (e.g., how many organisms are potentially present in a disaster zone); unfortunately, policy makers often have to make decisions in the face of large uncertainties (e.g., missing or incomplete information). Here, we have provided a statistically rigorous approach to derive reasonable estimates based on the best available information from different data sources. Our results also highlight the importance of thoughtful study design. Had we observed manatees in our sampled plots, we would have been able to estimate manatee density in the disaster area, which of course would have increased the value of information. However, even with no observations of manatees in the sampled plots, our study design could still be used to derive useful estimates, such as the upper estimate for the number of manatees potentially in the area. In contrast, traditional exhaustive search surveys could not have been used for that purpose. To conclude, our method is especially relevant for sparse data on species with low encounter rates, which occur when species are at low densities, are hard to detect, or have large ranges. We believe this approach will help improve future wildlife monitoring in disaster zones.
